# Nonlinear elasticity with limiting small strain for cracks subject to non-penetration

**DOI:** 10.1177/1081286516632380

**Published:** 2016-03-14

**Authors:** Hiromichi Itou, Victor A Kovtunenko, Kumbakonam R Rajagopal

**Affiliations:** Department of Mathematics, Tokyo University of Science, Tokyo, Japan; Institute for Mathematics and Scientific Computing, University of Graz, Graz, Austria; Lavrentyev Institute of Hydrodynamics, Siberian Division of the Russian Academy of Sciences, Novosibirsk, Russia; Department of Mechanical Engineering, Texas A&M University, TX, USA

**Keywords:** Nonlinear elasticity, limiting small strain, nonlinear crack with non-penetration, variational inequality, generalized solution, regularization, penalization

## Abstract

A major drawback of the study of cracks within the context of the linearized theory of elasticity is the inconsistency that one obtains with regard to the strain at a crack tip, namely it becoming infinite. In this paper we consider the problem within the context of an elastic body that exhibits limiting small strain wherein we are not faced with such an inconsistency. We introduce the concept of a non-smooth viscosity solution which is described by generalized variational inequalities and coincides with the weak solution in the smooth case. The well-posedness is proved by the construction of an approximation problem using elliptic regularization and penalization techniques.

## 1. Introduction

Recently, Rajagopal [1–3] introduced a new class of elastic bodies that are neither Cauchy elastic nor Green elastic that are defined through implicit constitutive relations. A special sub-class of these bodies are bodies wherein the Cauchy–Green strain is a function of the Cauchy stress, which when linearized within the context of the displacement gradient being small leads to the linearized strain being a function of the stress. That is, the theory allows for a nonlinear relationship between the linearized strain and the stress.

A further sub-class of these models possess the feature of the strain being limited by a certain value, irrespective of the value of the stress. Such a feature makes such constitutive relations possible candidates to describe the propagation of cracks and the fracture of brittle materials without having the deficiency of the classical linearized theory of elasticity, which predicts that the strains at the crack tip blow up as the inverse of the square root of the radial distance from the crack, violating the basic premise under which the linearized theory is derived. The implicit constitutive theory for elastic bodies has also been given a proper thermodynamic basis (see [4]).

Using such a strain limiting model Rajagopal and Walton [5] studied the problem of a crack subject to anti-plane shear and found that the strains are indeed bounded at the crack tip. Gou et al. [6] have studied the problem of a crack in a strain-limiting elastic body when the body is subject to plane strain and once again they find the strain to be bounded. Problems pertaining to notches in strain-limiting bodies have been studied by Kulvait et al. [7] and Bulicek et al. [8].

As the body can undergo only strains that can be bounded a priori, the strains are bounded uniformly over the solid, while the classical linearized elastic body has the drawback of unbounded even singular strains. The various issues concerning the mathematical difficulties related to the analysis of such models can be found in [9–11]. In particular, in [8], with the help of the anti-plane strain assumption the limiting small strain model was reduced to a problem akin to the minimal surface problem studied earlier for example in [12, Chapter 5] by employing dual variational methods. The modeling of fracture under the anti-plane simplification for solids with limiting small strain was carried out in [5].

In this paper, we investigate the well-posedness of the equations governing the equations of equilibrium for the problem of non-penetrating nonlinear cracks of elastic bodies that exhibit strain-limiting behavior.

With regard to studies that are relevant to this work within the context of fracture, see [13, 14] and related contact and damage mechanics studies in [15–19]. Nonlinear models of cracks satisfying the non-penetration condition were established as variational inequalities in the works [20–22] and developed further in [23, 24] for curvilinear cracks as well as in [25] for kinking cracks, in [26, 27] for frictional contact between the crack faces and in [28–30] for cohesive contact implying pseudo-monotone variational inequalities due to [31]. Recently, anti-cracks subject to non-penetration were treated; see for example [32] and references therein.

With respect to the contact conditions assumed between crack faces, limiting small strain models are able to provide the boundary trace of displacements at the crack faces. However, the lack of regularity of stresses does not allow one to determine accurately the normal stress at the boundary.

From the mathematical viewpoint, the standard existence theorems known for nonlinear elliptic problems, for example from [33], are not applicable to the limiting small strain model since the stress can be estimated a priori only in the non-reflexive *L^1^*-space. Therefore, we approximate the problem using elliptic regularization and the penalization of contact; see the regularization techniques in [34–36].

After taking the limit of the regularization parameter to zero using weak compactness and semi-continuity properties of the problem operator (see [37, Chapter 1]), in the limit we derive a non-smooth viscosity solution expressed by generalized variational inequalities, where the stresses can be determined only as bounded ${ℳ}^{1}$-measures. For smooth stresses and strains, these variational inequalities turn into the usual variational formulation of a weak solution of the problem.

## 2. Nonlinear crack problem with limiting small strain and non-penetration

Let Ω be a bounded domain in ${\mathbb{R}}^{d}$, d∈{2,3}, with Lipschitz boundary ∂Ω and the normal vector $n=(n_{1},\ldots ,n_{d})$, which is outward to Ω. We assume that $\partial \Omega =\bar{\Gamma_{N}}\cup \bar{\Gamma_{D}}$ consists of the Neumann boundary $\Gamma_{N}$ and the nonempty Dirichlet boundary $\Gamma_{D}\neq \emptyset$ parts. Let $\Gamma_{c}\subset \Omega$ be a crack, a (d−1)-dimensional oriented Lipschitz manifold which can be extended up to the external boundary ∂Ω such that Ω splits into two domains with Lipschitz boundaries. Depending on the chosen direction of the normal vector $n=(n_{1},\ldots ,n_{d})$ at $\Gamma_{c}$, the two crack faces, $\Gamma_{c}^{+}$ corresponding to the normal *n* inward to Ω, and its opposite $\Gamma_{c}^{-}$ with *n* outward to Ω, can be distinguished. Then $\Omega_{c}:=\Omega \backslash \Gamma_{c}$ denotes the domain with the crack and has the external boundary ∂Ω and the internal boundary $\bar{\Gamma_{c}^{+}}\cup \bar{\Gamma_{c}^{-}}$.

For a displacement vector $u(x)=(u_{1},\ldots ,u_{d})$ defined at spatial points $x=(x_{1},\ldots ,x_{d})$ over $\bar{\Omega_{c}}$ we set the *non-penetration condition*

(1a) $$(u{|}_{\Gamma_{c}^{+}}-u{|}_{\Gamma_{c}^{-}})\cdot n=:〚u〛\cdot n=〚u\cdot n〛\geq 0\;\mathrm{on}\Gamma_{c},$$

where the dot in $u\cdot n=u_{i}n_{i}$ implies the scalar product of vectors and the convention of summation over the repeated indexes i,j=1,…,d is used here and in what follows.

Given the body force $f=(f_{1},\ldots ,f_{d})\in L^{\max\{2,p\}}(\Omega_{c};{\mathbb{R}}^{d})$, the boundary traction $g=(g_{1},\ldots ,g_{d})\in L^{2}(\Gamma_{N};{\mathbb{R}}^{d})$, and the function $\Psi :\mathrm{Sym}({\mathbb{R}}^{d\times d})\to \mathrm{Sym}({\mathbb{R}}^{d\times d})$ defined over the symmetric matrices $\mathrm{Sym}({\mathbb{R}}^{d\times d})$, we set the *nonlinear problem with limiting small strain for crack subject to the condition* (1) in the weak form: for 1≤p<∞ and 1<p′≤∞ such that $\frac{1}{p}+\frac{1}{p\prime }=1$, find the displacement vector $u\in H^{1}(\Omega_{c};{\mathbb{R}}^{d})$, the strain tensor $e(u)=\{e_{\mathrm{ij}}(u){\}}_{i,j=1}^{d}\in L^{p\prime }(\Omega_{c};\mathrm{Sym}({\mathbb{R}}^{d\times d}))$, and the stress tensor $\sigma =\{\sigma_{\mathrm{ij}}{\}}_{i,j=1}^{d}\in L^{p}(\Omega_{c};\mathrm{Sym}({\mathbb{R}}^{d\times d}))$ such that

(1b) $$u=0\;\mathrm{on}\Gamma_{D},$$

(1c) $$\begin{array}{c} \int_{\Omega_{c}}\sigma :e(\bar{u}-u)\mathrm{dx}\geq \int_{\Omega_{c}}f\cdot (\bar{u}-u)\mathrm{dx}+\int_{\Gamma_{N}}g\cdot (\bar{u}-u)dS_{x}\;\mathrm{for all test functions}\\ \bar{u}\in H^{1}(\Omega_{c};{\mathbb{R}}^{d})\mathrm{such that}\;\bar{u}=0\;\mathrm{on}\;\Gamma_{D},〚\bar{u}\cdot n〛\geq 0\;\mathrm{on}\;\Gamma_{c},\mathrm{and}\;e(\bar{u})\in L^{p\prime }(\Omega_{c};\mathrm{Sym}({\mathbb{R}}^{d\times d})), \end{array}$$

(1d) $$\Psi (\sigma )=e(u):=\frac{1}{2}\{u_{i,j}+u_{j,i}{\}}_{i,j=1}^{d}\;\mathrm{in}\Omega_{c},$$

where $(u_{,1},\ldots ,u_{,d})=\nabla u$ stands for the gradient and the double dot in $\sigma :e(u)=\sigma_{\mathrm{ij}}e_{\mathrm{ij}}(u)$ implies the scalar product of matrices.

In (1c), the integrals are well defined as the duality between the dual spaces $L^{p}(\Omega_{c};\mathrm{Sym}({\mathbb{R}}^{d\times d}))$ and $L^{p\prime }(\Omega_{c};\mathrm{Sym}({\mathbb{R}}^{d\times d}))$ on the left-hand side, and $L^{2}(\Omega_{c};{\mathbb{R}}^{d})$ and $L^{2}(\Gamma_{N};{\mathbb{R}}^{d})$ on the right-hand side, respectively. In (1a) and (1c) the trace theorem for $u,\bar{u}\in H^{1}(\Omega_{c};{\mathbb{R}}^{d})\cap W^{1,p\prime }(\Omega_{c};{\mathbb{R}}^{d})$ provides the inclusion $u,\bar{u}\in H_{00}^{1/2}(\Gamma_{N};{\mathbb{R}}^{d})\cap W_{00}^{1/p,p\prime }(\Gamma_{N};{\mathbb{R}}^{d})$ and $〚u〛,〚\bar{u}〛\in H_{00}^{1/2}(\Gamma_{c};{\mathbb{R}}^{d})\cap W_{00}^{1/p,p\prime }(\Gamma_{c};{\mathbb{R}}^{d})$ in the Lions–Magenes space of functions which can be extended by zero; see for example [20, Section 1.1.7].

Below we outline the boundary value formulation of the variational inequality (1c).

We apply the following Green formula which holds for smooth $v\in C^{\infty }(\Omega_{c};{\mathbb{R}}^{d})$ that vanishes on $\Gamma_{D}$:

(2) $$\begin{array}{c} \int_{\Omega_{c}}(\sigma :e(v)+d\mathrm{iv}\sigma \cdot v)\mathrm{dx}=\int_{\Gamma_{N}}(\sigma \cdot n)\cdot vdS_{x}-\int_{\Gamma_{c}}〚(\sigma \cdot n)\cdot v〛dS_{x}, \end{array}$$

where the divergence $\mathrm{div}\sigma =(\sigma_{1j,j},\ldots ,\sigma_{\mathrm{dj},j})$ and the normal stress $\sigma \cdot n=(\sigma_{1j}n_{j},\ldots ,\sigma_{\mathrm{dj}}n_{j})$. Testing $\bar{u}=u\pm v$ with functions *v* vanishing on the Neumann boundary $\Gamma_{N}$ and the crack faces $\Gamma_{c}^{\pm }$, from (1c) and (2) we infer the common *equilibrium equation*:

(3a) $$\begin{array}{c} -\mathrm{div}\sigma =f\;\mathrm{in}\Omega_{c}. \end{array}$$

Moreover, by virtue of (2) and (3a) we define the linear functional over $W_{00}^{1/p,p\prime }(\Gamma_{N};{\mathbb{R}}^{d})\times W_{00}^{1/p,p\prime }(\Gamma_{c};{\mathbb{R}}^{d})$ through:

$$\begin{array}{c} {〈\sigma \cdot n,v〉}_{\Gamma_{N}\cup \Gamma_{c}}:=\int_{\Gamma_{N}}(\sigma \cdot n)\cdot vdS_{x}-\int_{\Gamma_{c}}(\sigma \cdot n)\cdot 〚v〛dS_{x}=\int_{\Omega_{c}}(\sigma :e(v)-f\cdot v)\mathrm{dx}, \end{array}$$

which is continuous after the use of the Cauchy–Schwarz inequality, the trace theorem with a constant $c_{1}>0$, holding for p′∈(1,∞); see [38, Theorem 3.54]:

$$\begin{array}{c} |{〈\sigma \cdot n,v〉}_{\Gamma_{N}\cup \Gamma_{c}}|\leq ∥\sigma {∥}_{L^{p}(\Omega_{c})}∥e(v){∥}_{L^{p\prime }(\Omega_{c})}+∥f{∥}_{L^{p}(\Omega_{c})}∥v{∥}_{L^{p\prime }(\Omega_{c})}\leq (∥\sigma {∥}_{L^{p}(\Omega_{c})}\\ +∥f{∥}_{L^{p}(\Omega_{c})})∥v{∥}_{W^{1,p\prime }(\Omega_{c})}\leq c_{1}(∥\sigma {∥}_{L^{p}(\Omega_{c})}+∥f{∥}_{L^{p}(\Omega_{c})})(∥v{∥}_{W_{00}^{1/p,p\prime }(\Gamma_{N})}+∥〚v〛{∥}_{W_{00}^{1/p,p\prime }(\Gamma_{c})}). \end{array}$$

Henceforth, the normal stress σ·n is defined well in the dual space $W^{-1/p\prime ,p}(\Gamma_{N};{\mathbb{R}}^{d})\times W^{-1/p\prime ,p}(\Gamma_{c};{\mathbb{R}}^{d})$ if p∈(1,∞), and in $H^{-1/2}(\Gamma_{N};{\mathbb{R}}^{d})\times H^{-1/2}(\Gamma_{c};{\mathbb{R}}^{d})$ if p∈[2,∞). In this case, the standard manipulation with test functions in (1c) using (1a), (2), and (3) results in the following complete system of boundary conditions:

(3b) $$\sigma \cdot n=g\;\mathrm{on}\Gamma_{N},$$

(3c) $$\begin{array}{c} \sigma \cdot n-((\sigma \cdot n)\cdot n)n=0,\;〚(\sigma \cdot n)\cdot n〛=0,\\ 〚u\cdot n〛\geq 0,\;(\sigma \cdot n)\cdot n\leq 0,\;((\sigma \cdot n)\cdot n)〚u\cdot n〛=0\;\mathrm{on}\;\;\Gamma_{c}. \end{array}$$

See See [20, Section 1.1.7] for details. Thus we have proved the following

**Proposition 1** *If p∈(1,∞) in the nonlinear crack problem (1), then the variational inequality (1c) implies the boundary value setting (3) with the normal stress $\sigma \cdot n\in W^{-1/p\prime ,p}(\Gamma_{N};{\mathbb{R}}^{d})\times W^{-1/p\prime ,p}(\Gamma_{c};{\mathbb{R}}^{d})$, where p′∈(1,∞) is such that $\frac{1}{p}+\frac{1}{p\prime }=1$.*

The key issue is the form of the function Ψ in the *constitutive equation* (1d). For comparison we refer to the known functions

$$\begin{array}{c} \mathrm{linearized elasticity}:\;\Psi (\sigma )=A\sigma ,\;A\in \mathrm{Sym}({\mathbb{R}}^{d\times d\times d\times d}),\\ \mathrm{power}-\mathrm{law hardening}:\;\Psi (\sigma )=A\sigma +\alpha |\sigma {|}^{s-2}\sigma ,\;\alpha \in {\mathbb{R}}_{+},\;s>2. \end{array}$$

See for example [39], where $|\sigma |=\sqrt{\sigma :\sigma }=\sqrt{\sigma_{\mathrm{ij}}\sigma_{\mathrm{ij}}}$ stands for the Frobenius matrix norm.

Following [3], we consider the limiting small strain function $\Psi :L^{1}(\Omega_{c};\mathrm{Sym}({\mathbb{R}}^{d\times d}))\mapsto L^{\infty }(\Omega_{c};\mathrm{Sym}$$\left({\mathbb{R}}^{d\times d})\right)$, which has the principal form

(4) $$\begin{array}{c} \Psi (\sigma )=\frac{\sigma }{2\mu {\left(1+\kappa |\sigma {|}^{s}\right)}^{1/s}},\;\mu ,\kappa ,s\in {\mathbb{R}}_{+}, \end{array}$$

where for simplicity we omit the spherical part depending on $tr\sigma =\sigma_{\mathrm{ii}}$. The main feature that one can immediately observe in (4) is the uniform bound $|\Psi (\sigma )|\leq \frac{1}{2\mu \kappa^{1/s}}$ which implies bounded strains $|e(u)|\leq \frac{1}{2\mu \kappa^{1/s}}$ according to (1d).

However, our consideration can be extended to the complete constitutive law; see [3]:

$$\begin{array}{c} \Psi (\sigma )=\alpha [1-\exp(\frac{-\lambda \mathrm{tr}\sigma }{{\left(1+\delta |\sigma {|}^{r}\right)}^{1/r}})]I+\frac{\sigma }{2\mu {\left(1+\kappa |\sigma {|}^{s}\right)}^{1/s}},\;\alpha ,\lambda ,\delta ,r,\mu ,\kappa ,s\in {\mathbb{R}}_{+}, \end{array}$$

where *I* stands for the d×d identity matrix, as a consequence of the positiveness and boundedness of the spherical part here. (This remark is due to discussion with J Málek.)

In the next section we present the key properties of the generating function (4), which will be used further in order to establish uniqueness, solvability, and a priori estimates of the reference problem (1).

### 2.1. Auxiliary results

Based on the estimation techniques from [8, 10] we present two auxiliary lemmas.

**Lemma 1** *The following estimate holds*:

(5) $$\begin{array}{c} {\left(1+\kappa |\sigma {|}^{s}\right)}^{1/s}\leq c_{s}(1+\kappa^{1/s}|\sigma |)\;\mathrm{with}\;c_{s}=2^{1/s-1}\mathrm{for}\;s\in (0,1)\mathrm{and}\;c_{s}=1\mathrm{for}s\geq 1. \end{array}$$

**Proof**. It suffices to prove the equivalent inequality

(6) $$\begin{array}{c} 1+\kappa |\sigma {|}^{s}\leq c_{s}^{s}{\left(1+\kappa^{1/s}|\sigma |\right)}^{s}\;\mathrm{with}\;c_{s}^{s}=2^{1-s}\mathrm{for}\;s\in (0,1)\mathrm{and}\;c_{s}^{s}=1\mathrm{for}\;s\geq 1 \end{array}$$

considering the three following cases. First, for s∈(0,1), Jensen’s inequality for concave $|\sigma {|}^{s}$ implies $\left(\frac{1}{2}+\frac{1}{2}\kappa^{1/s}|\sigma |{)}^{s}\geq \frac{1}{2}(1^{s}+\kappa |\sigma {|}^{s}\right)$ and (6) follows. Second, for s≥1 and $\kappa^{1/s}|\sigma |\in (0,1)$, the mean value theorem provides that there exists $\xi_{1}\in (1,1+\kappa^{1/s}|\sigma |)$ such that $(1+\kappa^{1/s}|\sigma |{)}^{s}-1^{s}=s\xi_{1}^{s-1}\kappa^{1/s}|\sigma |\geq \kappa^{1/s}|\sigma |\geq \kappa |\sigma {|}^{s}$. Third, for s≥1 and $\kappa^{1/s}|\sigma |\geq 1$, the mean value theorem again implies that there exists $\xi_{2}\in (\kappa^{1/s}|\sigma |,1+\kappa^{1/s}|\sigma |)$ such that $(1+\kappa^{1/s}|\sigma |{)}^{s}-\kappa |\sigma {|}^{s}=s\xi_{2}^{s-1}\geq (\kappa^{1/s}|\sigma |{)}^{s-1}\geq 1$. Thus, both the cases for s≥1 yield (6) and prove the lemma. □

**Lemma 2** (i) *In formula (4), Ψ is strictly monotone and continuous with the two-sided bounds*

(7a) $$\begin{array}{c} \frac{|\sigma^{1}-\sigma^{2}{|}^{2}}{2^{1+1/s}\mu [1+\kappa^{1+1/s}{\left(|\sigma^{1}|+|\sigma^{2}|\right)}^{1+s}]}\leq (\Psi (\sigma^{1})-\Psi (\sigma^{2})):(\sigma^{1}-\sigma^{2})\leq \frac{1}{\mu }|\sigma^{1}-\sigma^{2}{|}^{2}. \end{array}$$

(ii) Ψ*is bounded with the upper bound*

(7b) $$\begin{array}{c} |\Psi (\sigma )|\leq \frac{1}{2\mu }\min\{\frac{1}{\kappa^{1/s}},|\sigma |\}. \end{array}$$

(iii) $\int_{\Omega_{c}}\Psi (\sigma ):\sigma \mathrm{dx}$ *is coercive with the lower bound*

(7c) $$\begin{array}{c} \frac{1}{2\mu c_{s}\kappa^{1/s}}(\int_{\Omega_{c}}|\sigma |\mathrm{dx}-\frac{\left|\Omega \right|}{\kappa^{1/s}})\leq \int_{\Omega_{c}}\Psi (\sigma ):\sigma \mathrm{dx}. \end{array}$$

**Proof.** To prove (7a) in assertion (i) we employ the integral representation

(8) $$\begin{array}{c} \Psi (\sigma^{1})-\Psi (\sigma^{2})=\Psi (t\sigma^{1}+(1-t)\sigma^{2}){|}_{t=0}^{1}=\int_{0}^{1}\frac{d}{\mathrm{dt}}\Psi (t\sigma^{1}+(1-t)\sigma^{2})\mathrm{dt}, \end{array}$$

and compute the directional derivative of Ψ in (8) multiplied by $\sigma^{1}-\sigma^{2}$ as follows:

(9) $$\begin{array}{c} \frac{d}{\mathrm{dt}}\Psi (t\sigma^{1}+(1-t)\sigma^{2}):(\sigma^{1}-\sigma^{2})=\frac{1}{2\mu {\left[1+\kappa |t\sigma^{1}+(1-t)\sigma^{2}{|}^{s}\right]}^{1/s}}\\ \times \{|\sigma^{1}-\sigma^{2}{|}^{2}-|(t\sigma^{1}+(1-t)\sigma^{2}):(\sigma^{1}-\sigma^{2}{)|}^{2}\frac{\kappa |t\sigma^{1}+(1-t)\sigma^{2}{|}^{s-2}}{1+\kappa |t\sigma^{1}+(1-t)\sigma^{2}{|}^{s}}\} \end{array}$$

due to $\frac{d}{\mathrm{dt}}|t\sigma^{1}+(1-t)\sigma^{2}|=\frac{\left(t\sigma^{1}+(1-t)\sigma^{2}):(\sigma^{1}-\sigma^{2}\right)}{\left|t\sigma^{1}+(1-t)\sigma^{2}\right|}$.

Estimating from above the expression due to (9) yields:

$$\begin{array}{c} \left|\frac{d}{\mathrm{dt}}\Psi (t\sigma^{1}+(1-t)\sigma^{2})|\leq \frac{\left|\sigma^{1}-\sigma^{2}\right|}{2\mu {\left[1+\kappa |t\sigma^{1}+(1-t)\sigma^{2}{|}^{s}\right]}^{1/s}}\{1+\frac{\kappa |t\sigma^{1}+(1-t)\sigma^{2}{|}^{s}}{1+\kappa |t\sigma^{1}+(1-t)\sigma^{2}{|}^{s}}\right\}\\ \leq \frac{1}{\mu }|\sigma^{1}-\sigma^{2}|. \end{array}$$

Together with (8) the upper bound in (7a) follows, while the Cauchy–Schwarz inequality applied to the negative term on the right-hand side of (9) leads to the estimate from below:

(10) $$\begin{array}{c} \frac{d}{\mathrm{dt}}\Psi (t\sigma^{1}+(1-t)\sigma^{2}):(\sigma^{1}-\sigma^{2})\geq \frac{|\sigma^{1}-\sigma^{2}{|}^{2}}{2\mu {\left[1+\kappa |t\sigma^{1}+(1-t)\sigma^{2}{|}^{s}\right]}^{1/s}}\\ \times \{1-\frac{\kappa |t\sigma^{1}+(1-t)\sigma^{2}{|}^{s}}{1+\kappa |t\sigma^{1}+(1-t)\sigma^{2}{|}^{s}}\}=\frac{|\sigma^{1}-\sigma^{2}{|}^{2}}{2\mu {\left[1+\kappa |t\sigma^{1}+(1-t)\sigma^{2}{|}^{s}\right]}^{1+1/s}}. \end{array}$$

Using estimate (5) from Lemma 1 in (10) we obtain

$$\begin{array}{c} {\left[1+\kappa |t\sigma^{1}+(1-t)\sigma^{2}{|}^{s}\right]}^{1+1/s}\leq c_{s/(1+s)}[1+\kappa^{1+1/s}|t\sigma^{1}+(1-t)\sigma^{2}{|}^{1+s}]. \end{array}$$

Using $\left|t\sigma^{1}+(1-t)\sigma^{2}|\leq |\sigma^{1}|+|\sigma^{2}\right|$ and $c_{s/(1+s)}=2^{1/s}$, this concludes the establishment of the lower bound in (7a).

The upper bound (7b) in assertion (ii) follows straightforwardly from formula (4) for Ψ.

To get the lower bound (7c) in assertion (iii) we apply Young’s inequality together with (5):

$$\begin{array}{c} \frac{1}{\mu }\int_{\Omega_{c}}|\sigma |\mathrm{dx}=\frac{1}{\mu }\int_{\Omega_{c}}{\left(\frac{|\sigma {|}^{2}}{{\left(1+\kappa |\sigma {|}^{s}\right)}^{1/s}}\right)}^{1/2}{\left(1+\kappa |\sigma {|}^{s}\right)}^{1/2s}\mathrm{dx}\\ \leq \frac{c_{s}\kappa^{1/s}}{2\mu }\int_{\Omega_{c}}\frac{|\sigma {|}^{2}}{{\left(1+\kappa |\sigma {|}^{s}\right)}^{1/s}}\mathrm{dx}+\frac{1}{2\mu c_{s}\kappa^{1/s}}\int_{\Omega_{c}}{\left(1+\kappa |\sigma {|}^{s}\right)}^{1/s}\mathrm{dx}\\ \leq c_{s}\kappa^{1/s}\int_{\Omega_{c}}\Psi (\sigma ):\sigma \mathrm{dx}+\frac{1}{2\mu \kappa^{1/s}}\int_{\Omega_{c}}(1+\kappa^{1/s}|\sigma |)\mathrm{dx} \end{array}$$

and use $\int_{\Omega_{c}}\mathrm{dx}=:|\Omega_{c}|=|\Omega |$. The proof is complete. □

Based on Lemma 2, we obtain below an a priori estimate and uniqueness of the stresses in (1).

Further, we use the Korn–Poincaré inequality; see for example [40, Section 1]:

(11) $$c_{\mathrm{KP}}∥u{∥}_{H^{1}(\Omega_{c})}^{2}\leq \int_{\Omega_{c}}|e(u){|}^{2}\mathrm{dx}\;\mathrm{for}\;u\in H^{1}(\Omega_{c};{\mathbb{R}}^{d})\;\mathrm{such that}\;u=0\;\mathrm{on}\;\Gamma_{D},$$

and the solution $u^{E}\in H^{1}(\Omega_{c};{\mathbb{R}}^{d})$, $e(u^{E})\in L^{2}(\Omega_{c};\mathrm{Sym}({\mathbb{R}}^{d\times d}))$, and $\sigma^{E}\in L^{2}(\Omega_{c};\mathrm{Sym}({\mathbb{R}}^{d\times d}))$ of an auxiliary problem corresponding to the following equations of linearized elasticity:

(12a) $$u^{E}=0\;\mathrm{on}\Gamma_{D},$$

(12b) $$\begin{array}{c} \int_{\Omega_{c}}\sigma^{E}:e(\bar{u})\mathrm{dx}=\int_{\Omega_{c}}f\cdot \bar{u}\mathrm{dx}+\int_{\Gamma_{N}}g\cdot \bar{u}dS_{x}\\ \mathrm{for all test functions}\;\bar{u}\in H^{1}(\Omega_{c};{\mathbb{R}}^{d})\;\mathrm{such that}\;\bar{u}=0\;\mathrm{on}\;\Gamma_{D}, \end{array}$$

(12c) $$\sigma^{E}=e(u^{E})\;\mathrm{in}\Omega_{c}.$$

**Proposition 2.** *The stress tensor σ for the nonlinear crack problem (1) is unique, and the following a priori estimate holds*:

(13) $$\begin{array}{c} ∥\sigma {∥}_{L^{1}(\Omega_{c})}\leq \frac{\left|\Omega \right|}{\kappa^{1/s}}+c_{s}∥\sigma^{E}{∥}_{L^{1}(\Omega_{c})}, \end{array}$$

*where* |Ω| *stands for the Hausdorff measure of* Ω *in* ${\mathbb{R}}^{d}$, *and the elastic stress tensor* $\sigma^{E}$ *is given in (12)*.

**Proof.** In order to prove the uniqueness of the stress tensor, we assume two different solutions $\left(u^{k},e(u^{k}),\sigma^{k}\right)$ for k=1,2 to the problem (1), that is:

(14a) $$〚u^{k}\cdot n〛\geq 0\;\mathrm{on}\Gamma_{c},\;u^{k}=0\;\mathrm{on}\Gamma_{D},$$

(14b) $$\begin{array}{c} \int_{\Omega_{c}}\sigma^{k}:e({\bar{u}}^{k}-u^{k})\mathrm{dx}\geq \int_{\Omega_{c}}f\cdot ({\bar{u}}^{k}-u^{k})\mathrm{dx}+\int_{\Gamma_{N}}g\cdot ({\bar{u}}^{k}-u^{k})dS_{x}\\ \mathrm{for all test functions}\;{\bar{u}}^{k}\in H^{1}(\Omega_{c};{\mathbb{R}}^{d})\;\mathrm{such that}\;{\bar{u}}^{k}=0\;\mathrm{on}\;\Gamma_{D},〚{\bar{u}}^{k}\cdot n〛\geq 0\;\mathrm{on}\;\Gamma_{c},\\ \mathrm{and}\;e({\bar{u}}^{k})\in L^{p\prime }(\Omega_{c};\mathrm{Sym}({\mathbb{R}}^{d\times d})), \end{array}$$

(14c) $$\Psi (\sigma^{k})=e(u^{k})\;\mathrm{in}\;\;\Omega_{c}.$$

Testing ${\bar{u}}^{k}=u^{2}$ as k=1 and ${\bar{u}}^{k}=u^{1}$ as k=2 in (14b), summing these inequalities, and using the identities (14c) subsequently for k=1,2, it follows that

$$\begin{array}{c} 0\geq \int_{\Omega_{c}}(\sigma^{1}-\sigma^{2}):e(u^{1}-u^{2})\mathrm{dx}=\int_{\Omega_{c}}(\Psi (\sigma^{1})-\Psi (\sigma^{2})):(\sigma^{1}-\sigma^{2})\mathrm{dx}>0 \end{array}$$

for $\sigma^{1}\neq \sigma^{2}$ due to the strict monotony of Ψ established in Lemma 2, which is a contradiction and proves $\sigma^{1}=\sigma^{2}$.

To obtain the a priori estimate (13), we employ the auxiliary equation (12b) and rewrite (1c) equivalently as

(15) $$\begin{array}{c} \int_{\Omega_{c}}(\sigma -\sigma^{E}):e(\bar{u}-u)\mathrm{dx}\geq 0\;\mathrm{for all test functions}\;\bar{u}\in H^{1}(\Omega_{c};{\mathbb{R}}^{d})\\ \mathrm{such that}\;\bar{u}=0\;\mathrm{on}\;\Gamma_{D},〚\bar{u}\cdot n〛\geq 0\;\mathrm{on}\;\Gamma_{c},\mathrm{and}\;e(\bar{u})\in L^{p\prime }(\Omega_{c};\mathrm{Sym}({\mathbb{R}}^{d\times d})). \end{array}$$

Testing $\bar{u}=\mathrm{tu}$ with arbitrary t>0 due to the cone property of the constraint (1a) and using the identity (1d), from (15) it follows that the following equality holds:

(16) $$\begin{array}{c} 0=\int_{\Omega_{c}}(\sigma -\sigma^{E}):e(u)\mathrm{dx}=\int_{\Omega_{c}}\Psi (\sigma ):(\sigma -\sigma^{E})\mathrm{dx}. \end{array}$$

After application of the lower bound (7c) and the upper bound (7b) from Lemma 2 together with the Cauchy–Schwarz inequality to the equation (16) we proceed as follows:

$$\begin{array}{c} \frac{1}{2\mu c_{s}\kappa^{1/s}}(\int_{\Omega_{c}}|\sigma |\mathrm{dx}-\frac{\left|\Omega \right|}{\kappa^{1/s}})\leq \int_{\Omega_{c}}\Psi (\sigma ):\sigma \mathrm{dx}\leq |\Psi (\sigma )|\int_{\Omega_{c}}|\sigma^{E}|\mathrm{dx}\leq \frac{1}{2\mu \kappa^{1/s}}\int_{\Omega_{c}}|\sigma^{E}|\mathrm{dx}. \end{array}$$

This implies the estimate (13) and finishes the proof.

We emphasize that the auxiliary function $\sigma^{E}$ is introduced for convenience and its norm employed in the estimates (13) and (18a) can be evaluated by the data $∥f{∥}_{L^{2}(\Omega_{c})}+∥g{∥}_{L^{2}(\Gamma_{N})}$.

From Proposition 2 we conclude that the stresses in (13) are estimated only in the non-reflexive $L^{1}$-space, which is not weakly compact. Therefore, to investigate solvability of the nonlinear crack problem (1), in the next section we construct an elliptic regularization of the equilibrium and constitutive relations (1c) and (1d). To regularize the unilateral constraint (1a) we utilize penalization.

### 2.2. Problem approximation by elliptic and penalty regularization

For a fixed regularization parameter ε>0, we set the regularized crack problem with limiting small strain and penalization in the weak form: find the displacement vector $u^{\varepsilon }\in H^{1}(\Omega_{c};{\mathbb{R}}^{d})$, the strain tensor $e(u^{\varepsilon })\in L^{2}(\Omega_{c};\mathrm{Sym}({\mathbb{R}}^{d\times d}))$, and the stress tensor $\sigma^{\varepsilon }\in L^{2}(\Omega_{c};\mathrm{Sym}({\mathbb{R}}^{d\times d}))$ such that

(17a) $$u^{\varepsilon }=0\;\mathrm{on}\;\Gamma_{D},$$

(17b) $$\begin{array}{c} \int_{\Omega_{c}}(\varepsilon e(u^{\varepsilon })+\sigma^{\varepsilon }):e(\bar{u})\mathrm{dx}+\frac{1}{\varepsilon }\int_{\Gamma_{c}}\min\{0,〚u^{\varepsilon }\cdot n〛\}〚\bar{u}\cdot n〛dS_{x}=\int_{\Omega_{c}}f\cdot \bar{u}\mathrm{dx}\\ +\int_{\Gamma_{N}}g\cdot \bar{u}\;dS_{x}\;\mathrm{for all test functions}\;\bar{u}\in H^{1}(\Omega_{c};{\mathbb{R}}^{d})\;\mathrm{such that}\;\bar{u}=0\;\mathrm{on}\;\Gamma_{D}, \end{array}$$

(17c) $$\varepsilon \sigma^{\varepsilon }+\Psi (\sigma^{\varepsilon })=e(u^{\varepsilon })\;\mathrm{in}\;\;\Omega_{c}.$$

We note that regularized strains in (17c) are not uniformly bounded, and they are redundant as unknowns from the system (17) since $e(u^{\varepsilon })=\frac{1}{2}\{u_{i,j}^{\varepsilon }+u_{j,i}^{\varepsilon }{\}}_{i,j=1}^{d}$.

**Theorem 1.** *For fixed $\varepsilon \in (0,\varepsilon_{0})$, there exists the unique solution of the regularized crack problem (17) that satisfies the following a priori estimates uniformly in ε*:

(18a) $$\begin{array}{c} \varepsilon c_{\mathrm{KP}}∥u^{\varepsilon }{∥}_{H^{1}(\Omega_{c})}^{2}+\frac{\varepsilon }{2}∥\sigma^{\varepsilon }{∥}_{L^{2}(\Omega_{c})}^{2}+\frac{1}{2\mu c_{s}\kappa^{1/s}}∥\sigma^{\varepsilon }{∥}_{L^{1}(\Omega_{c})}+\frac{1}{\varepsilon }∥\min\{0,〚u^{\varepsilon }\cdot n〛\}{∥}_{L^{2}(\Gamma_{c})}^{2}\\ \leq \frac{\varepsilon_{0}}{2}∥\sigma^{E}{∥}_{L^{2}(\Omega_{c})}^{2}+\frac{1}{2\mu \kappa^{1/s}}(\frac{\left|\Omega \right|}{c_{s}\kappa^{1/s}}+∥\sigma^{E}{∥}_{L^{1}(\Omega_{c})})=:c_{\mathrm{RHS}}, \end{array}$$

(18b) $$c_{\mathrm{KP}}∥u^{\varepsilon }{∥}_{H^{1}(\Omega_{c})}^{2}\leq ∥e(u^{\varepsilon }){∥}_{L^{2}(\Omega_{c})}^{2}\leq \frac{\left|\Omega \right|}{2(\mu \kappa^{1/s}{)}^{2}}+4\varepsilon_{0}c_{\mathrm{RHS}},$$

*where the elastic stress tensor* $\sigma^{E}$ *is given in (12)*.

**Proof.** For fixed ε, the existence of the unique solution to problem (17) follows from the Browder–Minty theorem (see e.g [41, Theorem 2.18]), since the high-order terms in (17) are linear and the nonlinear terms are monotone, continuous (hence, demi-continuous), coercive, and bounded. Indeed, these properties of the penalty operator $\frac{1}{\varepsilon }\min\{0,〚u^{\varepsilon }\cdot n〛\}\in L^{2}(\Gamma_{c};\mathbb{R})$ are well known (see e.g. [20, Section 1.3.2]) while the properties of the nonlinear term Ψ are accounted for by Lemma 2.

To derive the a priori estimate (18a), with the help of the auxiliary elastic equation (12b) we rewrite the variational equation (17b) equivalently as

$$\begin{array}{c} \int_{\Omega_{c}}(\varepsilon e(u^{\varepsilon })+\sigma^{\varepsilon }-\sigma^{E}):e(\bar{u})\mathrm{dx}+\frac{1}{\varepsilon }\int_{\Gamma_{c}}\min\{0,〚u^{\varepsilon }\cdot n〛\}〚\bar{u}\cdot n〛dS_{x}=0\\ \mathrm{for all test functions}\;\bar{u}\in H^{1}(\Omega_{c};{\mathbb{R}}^{d})\;\mathrm{such that}\;\bar{u}=0\;\mathrm{on}\;\Gamma_{D}. \end{array}$$

Here we insert the test function $\bar{u}=u^{\varepsilon }$, replace $e(u^{\varepsilon })$ with the help of (17c):

$$\begin{array}{c} \int_{\Omega_{c}}(\varepsilon |e(u^{\varepsilon }{)|}^{2}+(\sigma^{\varepsilon }-\sigma^{E}):(\varepsilon \sigma^{\varepsilon }+\Psi (\sigma^{\varepsilon })))\mathrm{dx}+\frac{1}{\varepsilon }\int_{\Gamma_{c}}\min\{0,〚u^{\varepsilon }\cdot n〛\}〚u^{\varepsilon }\cdot n〛dS_{x}=0, \end{array}$$

rearrange

$$\begin{array}{c} (\sigma^{\varepsilon }-\sigma^{E}):(\varepsilon \sigma^{\varepsilon }+\Psi (\sigma^{\varepsilon }))=\varepsilon |\sigma^{\varepsilon }{|}^{2}+\Psi (\sigma^{\varepsilon }):\sigma^{\varepsilon }-\sigma^{E}:(\varepsilon \sigma^{\varepsilon }+\Psi (\sigma^{\varepsilon })), \end{array}$$

and use the identity

(19) $$\begin{array}{c} \min\{0,〚u^{\varepsilon }\cdot n〛\}〚u^{\varepsilon }\cdot n〛=\min\{0,〚u^{\varepsilon }\cdot n〛\}(\max\{0,〚u^{\varepsilon }\cdot n〛\}+\min\{0,〚u^{\varepsilon }\cdot n〛\})\\ =(\min\{0,〚u^{\varepsilon }\cdot n]{]\})}^{2}\geq 0 \end{array}$$

to get

(20) $$\begin{array}{c} \int_{\Omega_{c}}(\varepsilon |e(u^{\varepsilon }{)|}^{2}+\varepsilon |\sigma^{\varepsilon }{|}^{2}+\Psi (\sigma^{\varepsilon }):\sigma^{\varepsilon })\mathrm{dx}+\frac{1}{\varepsilon }\int_{\Gamma_{c}}{\left(\min\{0,〚u^{\varepsilon }\cdot n〛\}\right)}^{2}dS_{x}\\ =\int_{\Omega_{c}}\sigma^{E}:(\varepsilon \sigma^{\varepsilon }+\Psi (\sigma^{\varepsilon }))\mathrm{dx}\leq \frac{\varepsilon }{2}\int_{\Omega_{c}}|\sigma^{\varepsilon }{|}^{2}\mathrm{dx}+\frac{\varepsilon }{2}\int_{\Omega_{c}}|\sigma^{E}{|}^{2}\mathrm{dx}+|\Psi (\sigma^{\varepsilon })|\int_{\Omega_{c}}|\sigma^{E}|\mathrm{dx}, \end{array}$$

where we have applied the Young and Cauchy–Schwarz inequalities on the right-hand side of (20). Estimating the left-hand side of (20) from below with the help of the Korn–Poincare inequality (11) and the lower bound (7c) in Lemma 2, as well as the right-hand side of (20) from above using the uniform upper bound in (7b), we conclude with the estimate (18a).

Therefore, the subsequent squaring and integration over $\Omega_{c}$ of (17c) implies

$$\begin{array}{c} \int_{\Gamma_{c}}|e(u^{\varepsilon }{)|}^{2}\mathrm{dx}\leq 2\int_{\Omega_{c}}(|\Psi (\sigma^{\varepsilon }{)|}^{2}+{\left(\varepsilon |\sigma^{\varepsilon }|\right)}^{2})\mathrm{dx}\leq 2|\Psi (\sigma^{\varepsilon }{)|}^{2}\int_{\Omega_{c}}\mathrm{dx}+2\varepsilon^{2}\int_{\Omega_{c}}|\sigma^{\varepsilon }{|}^{2}\mathrm{dx} \end{array}$$

and leads to the estimate (18b) after using (7b), (18a), and the Korn–Poincare inequality (11). This completes the proof. □

Based on Theorem 1, next we pass $\varepsilon ↘0^{+}$ to the limit in (17).

Since $L^{1}(\Omega_{c})$ is not reflexive (hence, not weakly compact) we employ the embedding $L^{1}(\Omega_{c})=L^{1}(\Omega )↪{ℳ}^{1}(\Omega )$ in the space of bounded measures, which is dual to the space $C_{c}(\Omega )$ of continuous functions with compact support in Ω, such that

(21) $$c_{BM}∥\sigma {∥}_{{ℳ}^{1}(\Omega )}\leq ∥\sigma {∥}_{L^{1}(\Omega )}$$

(see e.g. [40, Chapter 3, Section 2]), and prove the existence theorem.

**Theorem 2** (i) *There exists a generalized solution*

(22) $$u\in H^{1}(\Omega_{c};{\mathbb{R}}^{d}),\;e(u)\in L^{2}(\Omega_{c};\mathrm{Sym}({\mathbb{R}}^{d\times d})),\;\sigma \in {ℳ}^{1}(\Omega ;\mathrm{Sym}({\mathbb{R}}^{d\times d})),$$

*determined as a weak accumulation point of the solutions of the regularized crack problems (17) as $\varepsilon ↘0^{+}$, which satisfies the following generalized variational problem:*

(23a) $$〚u\cdot n〛\geq 0\;\mathrm{on}\Gamma_{c},$$

(23b) $$u=0\;\mathrm{on}\Gamma_{D},$$

(23c) $$\begin{array}{c} \int_{\Omega }\sigma :e(\bar{u})\mathrm{dx}\geq \int_{\Omega_{c}}f\cdot \bar{u}\mathrm{dx}+\int_{\Gamma_{N}}g\cdot \bar{u}\;dS_{x}\;\mathrm{for all test functions}\;\bar{u}\in H^{1}(\Omega_{c};{\mathbb{R}}^{d})\\ \mathrm{such that}\;\bar{u}=0\;\mathrm{on}\;\Gamma_{D},〚\bar{u}\cdot n〛\geq 0\;\mathrm{on}\;\Gamma_{c},\mathrm{and}\;e(\bar{u})\in C_{c}(\Omega ;\mathrm{Sym}({\mathbb{R}}^{d\times d})), \end{array}$$

(23d) $$\begin{array}{c} \int_{\Omega }(\sigma -\bar{\sigma }):\Psi (\bar{\sigma })\mathrm{dx}+\int_{\Omega_{c}}e(u):\bar{\sigma }\mathrm{dx}\leq \int_{\Omega_{c}}f\cdot u\mathrm{dx}+\int_{\Gamma_{N}}g\cdot udS_{x}\\ \mathrm{for all test functions}\;\bar{\sigma }\in C_{c}(\Omega ;\mathrm{Sym}({\mathbb{R}}^{d\times d})). \end{array}$$

*The integrals over* Ω *in (23c) and (23d) are well defined as the duality between* ${ℳ}^{1}(\Omega )$ *and* $C_{c}(\Omega )$.

(ii) *If the stresses* $\sigma \in L^{p}(\Omega_{c};\mathrm{Sym}({\mathbb{R}}^{d\times d}))$ *and the strains* $e(u)\in L^{p\prime }(\Omega_{c};\mathrm{Sym}({\mathbb{R}}^{d\times d}))$ *are extra regular, where* 1≤p<∞ *and* 1<p′≤∞ *are such that* $\frac{1}{p}+\frac{1}{p\prime }=1$, *then the generalized variational inequalities (23c) and (23d) turn into the weak relations (1c) and (1d), and the triple* (u,e(u),σ) *from (22) solves the reference crack problem (1) subject to non-penetration and limiting small strains. Moreover, then* $e(u)\in L^{\infty }(\Omega_{c};\mathrm{Sym}({\mathbb{R}}^{d\times d}))$ *due to (1d)*.

**Proof.** We start with the assertion (i). Indeed, for $\varepsilon ↘0^{+}$, from the uniform estimates (18) and (21) in the standard way we infer a weak accumulation point (22) of a subsequence, still marked by ε, such that

(24a) $$u^{\varepsilon }⇀u\;\mathrm{weakly in}H^{1}(\Omega_{c};{\mathbb{R}}^{d}),\;e(u^{\varepsilon })⇀e(u)\;\mathrm{weakly in}L^{2}(\Omega_{c};\mathrm{Sym}({\mathbb{R}}^{d\times d})),$$

(24b) $$\min\{0,〚u^{\varepsilon }\cdot n〛\}⇀0\;\mathrm{weakly in}L^{2}(\Gamma_{c};\mathbb{R}),$$

(24c) $$\varepsilon \sigma^{\varepsilon }⇀0\;\mathrm{weakly in}L^{2}(\Omega_{c};\mathrm{Sym}({\mathbb{R}}^{d\times d})),$$

(24d) $$\sigma^{\varepsilon }⇀\sigma \;⋆-\mathrm{weakly in}{ℳ}^{1}(\Omega ;\mathrm{Sym(}{\mathbb{R}}^{d\times d}\mathrm{))}.$$

Due to the convergences (24a), from (17a) the Dirichlet condition (23b) follows, and the convergence (24b) leads to the non-penetration condition (23a).

For a test function $\bar{u}\in H^{1}(\Omega_{c};{\mathbb{R}}^{d})$ such that $\bar{u}=0$ on $\Gamma_{D}$, $〚\bar{u}\cdot n〛\geq 0$ on $\Gamma_{c}$, and $e(\bar{u})\in C_{c}(\Omega ;\mathrm{Sym}({\mathbb{R}}^{d\times d}))$, from (17b) we have

(25) $$\begin{array}{c} \int_{\Omega_{c}}(\varepsilon e(u^{\varepsilon })+\sigma^{\varepsilon }):e(\bar{u})\mathrm{dx}-\int_{\Omega_{c}}f\cdot \bar{u}\mathrm{dx}-\int_{\Gamma_{N}}g\cdot \bar{u}dS_{x}\\ =-\frac{1}{\varepsilon }\int_{\Gamma_{c}}\min\{0,〚u^{\varepsilon }\cdot n〛\}〚\bar{u}\cdot n〛dS_{x}\geq 0. \end{array}$$

Therefore, passing $\varepsilon ↘0^{+}$ in (25) the convergences (24a) and (24d) lead to the inequality (23c).

In order to pass to the limit in the nonlinear equation (17c) we apply the so-called Minty’s trick; see e.g. [41, Lemma 2.13].

For a test function $\bar{\sigma }\in C_{c}(\Omega ;\mathrm{Sym}({\mathbb{R}}^{d\times d}))$, we multiply the equation (17c) by $\sigma^{\varepsilon }-\bar{\sigma }$, integrate it over $\Omega_{c}$:

$$\begin{array}{c} \int_{\Omega_{c}}(\varepsilon \sigma^{\varepsilon }+\Psi (\sigma^{\varepsilon })-e(u^{\varepsilon })):(\sigma^{\varepsilon }-\bar{\sigma })\mathrm{dx}=0, \end{array}$$

then add equation (17b) with the test functions $\bar{u}=u^{\varepsilon }$:

$$\begin{array}{c} \int_{\Omega_{c}}(\varepsilon |e(u^{\varepsilon }{)|}^{2}+\sigma^{\varepsilon }:e(u^{\varepsilon }))\mathrm{dx}+\frac{1}{\varepsilon }\int_{\Gamma_{c}}\min\{0,〚u^{\varepsilon }\cdot n〛\}〚u^{\varepsilon }\cdot n〛dS_{x}\\ =\int_{\Omega_{c}}f\cdot u^{\varepsilon }\mathrm{dx}+\int_{\Gamma_{N}}g\cdot u^{\varepsilon }dS_{x}, \end{array}$$

and get due to (19), and similarly due to (20), that

(26) $$\begin{array}{c} \int_{\Omega_{c}}(\varepsilon |e(u^{\varepsilon }{)|}^{2}+(\varepsilon \sigma^{\varepsilon }+\Psi (\sigma^{\varepsilon })):(\sigma^{\varepsilon }-\bar{\sigma })+e(u^{\varepsilon }):\bar{\sigma })\mathrm{dx}\\ -\int_{\Omega_{c}}f\cdot u^{\varepsilon }\mathrm{dx}-\int_{\Gamma_{N}}g\cdot u^{\varepsilon }dS_{x}=-\frac{1}{\varepsilon }\int_{\Gamma_{c}}{\left(\min\{0,〚u^{\varepsilon }\cdot n〛\}\right)}^{2}dS_{x}\leq 0. \end{array}$$

Using the monotony of the second term on the left-hand side of (26) (see (7a)) it follows that

(27) $$\begin{array}{c} \int_{\Omega_{c}}(\varepsilon |e(u^{\varepsilon }{)|}^{2}+(\varepsilon \bar{\sigma }+\Psi (\bar{\sigma })):(\sigma^{\varepsilon }-\bar{\sigma })+e(u^{\varepsilon }):\bar{\sigma })\mathrm{dx}\leq \int_{\Omega_{c}}f\cdot u^{\varepsilon }\mathrm{dx}+\int_{\Gamma_{N}}g\cdot u^{\varepsilon }dS_{x}. \end{array}$$

Since the first term on the left-hand side of (27) implies the $L^{2}$-norm, which is weakly lower semi-continuous (see e.g. [37, Remark 2.3]), passing to the limit as $\varepsilon ↘0^{+}$ due to the convergences (24a), (24c), and (24d), we obtain the inequality (23d).

Now we prove the assertion (ii). Let the stress σ and strain e(u) in the generalized problem (23) possess the extra regularity $\sigma \in L^{p}(\Omega_{c};\mathrm{Sym}({\mathbb{R}}^{d\times d}))$ and $e(u)\in L^{p\prime }(\Omega_{c};\mathrm{Sym}({\mathbb{R}}^{d\times d}))$ with 1≤p<∞ and 1<p′≤∞ such that $\frac{1}{p}+\frac{1}{p\prime }=1$. Since the boundary conditions (23a) and (23b) coincide with (1a) and (1b), we will show that the variational inequality (1c) and the identity (1d) hold in this case.

In the smooth case, using the fact that the space $C_{c}(\Omega )$ is dense in $L^{p\prime }(\Omega_{c})$ (see e.g. [38, Theorem 1.91]) from (23c) it follows that

(28) $$\begin{array}{c} \int_{\Omega_{c}}\sigma :e(\bar{u})\mathrm{dx}\geq \int_{\Omega_{c}}f\cdot \bar{u}\mathrm{dx}+\int_{\Gamma_{N}}g\cdot \bar{u}dS_{x}\;\mathrm{for all test functions}\;\bar{u}\in H^{1}(\Omega_{c};{\mathbb{R}}^{d})\\ \mathrm{such that}\;\bar{u}=0\mathrm{on}\Gamma_{D},〚\bar{u}\cdot n〛\geq 0\;\mathrm{on}\;\Gamma_{c},\mathrm{and}\;e(\bar{u})\in L^{p\prime }(\Omega_{c};\mathrm{Sym}({\mathbb{R}}^{d\times d})), \end{array}$$

where the integral on the left-hand side of (28) is understood as the duality between $L^{p}(\Omega_{c};\mathrm{Sym}({\mathbb{R}}^{d\times d}))$ and $L^{p\prime }(\Omega_{c};\mathrm{Sym}({\mathbb{R}}^{d\times d}))$. Similarly, $C_{c}(\Omega )$ is dense in $L^{2}(\Omega_{c})$, and $\Psi (\bar{\sigma })\in L^{\infty }(\Omega_{c};\mathrm{Sym}({\mathbb{R}}^{d\times d}))↪L^{p\prime }(\Omega_{c};\mathrm{Sym}({\mathbb{R}}^{d\times d}))$ for all finite p′. Therefore, from (23d) we conclude that

(29) $$\begin{array}{c} \int_{\Omega_{c}}(\Psi (\bar{\sigma }):(\sigma -\bar{\sigma })+e(u):\bar{\sigma })\mathrm{dx}\leq \int_{\Omega_{c}}f\cdot u\mathrm{dx}+\int_{\Gamma_{N}}g\cdot udS_{x}\\ \mathrm{for all test functions}\;\bar{\sigma }\in L^{p}(\Omega_{c};\mathrm{Sym}({\mathbb{R}}^{d\times d})), \end{array}$$

where the integral on the left-hand side of (29) is defined well as the duality between $L^{p\prime }(\Omega_{c};\mathrm{Sym}({\mathbb{R}}^{d\times d}))$ and $L^{p}(\Omega_{c};\mathrm{Sym}({\mathbb{R}}^{d\times d}))$.

The smooth solution itself can be taken as the test functions $\bar{u}=u$ and $\bar{\sigma }=\sigma$ in (28) and (29):

$$\begin{array}{c} \begin{array}{c} \int_{\Omega_{c}}\sigma :e(u)\mathrm{dx}\geq \int_{\Omega_{c}}f\cdot u\mathrm{dx}+\int_{\Gamma_{N}}g\cdot udS_{x}, \end{array}\\ \begin{array}{c} \int_{\Omega_{c}}e(u):\sigma \mathrm{dx}\leq \int_{\Omega_{c}}f\cdot u\mathrm{dx}+\int_{\Gamma_{N}}g\cdot udS_{x}, \end{array} \end{array}$$

which results in the equality

(30) $$\begin{array}{c} \int_{\Omega_{c}}\sigma :e(u)\mathrm{dx}=\int_{\Omega_{c}}f\cdot u\mathrm{dx}+\int_{\Gamma_{N}}g\cdot udS_{x}. \end{array}$$

Together (28) and (30) are equivalent to the variational inequality (1c).

Subtracting equation (30) from (29) we have

$$\begin{array}{c} \int_{\Omega_{c}}(\Psi (\bar{\sigma })-e(u)):(\sigma -\bar{\sigma })\mathrm{dx}\leq 0. \end{array}$$

After plugging in $\bar{\sigma }=\sigma \pm t\tilde{\sigma }$ with arbitrary t>0 and $\tilde{\sigma }\in L^{p}(\Omega_{c};\mathrm{Sym}({\mathbb{R}}^{d\times d}))$, and dividing the result by *t*, we have

(31) $$\begin{array}{c} \mp \int_{\Omega_{c}}(\Psi (\sigma \pm t\tilde{\sigma })-e(u)):\tilde{\sigma }\mathrm{dx}\leq 0\;\mathrm{for all test functions}\;\tilde{\sigma }\in L^{p}(\Omega_{c};\mathrm{Sym}({\mathbb{R}}^{d\times d})). \end{array}$$

Passing $t↘0^{+}$ in (31) due to the demi-continuity of Ψ, which follows from the continuity of Ψ stated in Lemma 2, we obtain the equality (1d). The proof is complete.

**Remark 1.** *In assertion (ii) of Theorem 2, if the stresses $\sigma \in L^{p}(\Omega_{c};\mathrm{Sym}({\mathbb{R}}^{d\times d}))$ with p>1, then the normal stress is defined at the boundary due to Proposition 1.*

To conclude this section we summarize the main result: Theorem 1 and Theorem 2 together prove that the regularized crack problem (17) implies a constructive approximation of the nonlinear problem (1) with limiting small strain for cracks subject to non-penetration. This approximation is useful for analysis as well as possibly being reasonable for numerical computation.

In the last section we discuss in-plane and anti-plane simplifications of the model.

## 3. Discussion: Plane strain and anti-plane strain problems with limiting small strain for cracks subject to non-penetration

In the physical spatial setting of the nonlinear crack problem (1) we have d=3. Let the reference domain in ${\mathbb{R}}^{3}$ be Ω×ℝ, with the boundary ∂Ω×ℝ and the crack $\Gamma_{c}\times \mathbb{R}$ possessing the normal vector $n=(n_{1},n_{2},0)$, such that its plane cross-section $\Omega \backslash \Gamma_{c}=:\Omega_{c}\subset {\mathbb{R}}^{2}$ identifies the plane domain containing the crack.

First, if we make the plane strain assumption for the displacement, stresses and strains, respectively:

$$\begin{array}{c} u(x_{1},x_{2})=(u_{1},u_{2},0),\;e(u)=\left[\begin{array}{ccc} u_{1,1} & \frac{u_{1,2}+u_{2,1}}{2} & 0\\ \frac{u_{1,2}+u_{2,1}}{2} & u_{2,2} & 0\\ 0 & 0 & 0 \end{array}\right],\;\sigma =\left[\begin{array}{ccc} \sigma_{11} & \sigma_{12} & 0\\ \sigma_{12} & \sigma_{22} & 0\\ 0 & 0 & 0 \end{array}\right], \end{array}$$

then the plane problem with limiting small strain for cracks subject to non-penetration implies system (1) for d=2.

Second, we employ the anti-plane strain assumption:

(32) $$\begin{array}{c} u(x_{1},x_{2})=(0,0,u_{3}),\;e(u)=\left[\begin{array}{ccc} 0 & 0 & \frac{u_{3,1}}{2}\\ 0 & 0 & \frac{u_{3,2}}{2}\\ \frac{u_{3,1}}{2} & \frac{u_{3,2}}{2} & 0 \end{array}\right],\;\sigma =\left[\begin{array}{ccc} 0 & 0 & \sigma_{13}\\ 0 & 0 & \sigma_{23}\\ \sigma_{13} & \sigma_{23} & 0 \end{array}\right]. \end{array}$$

In order to model the three-dimensional nature of contact, we suggest resetting the inclined vector in ${\mathbb{R}}^{3}$,

(33) $$\frac{1}{\sqrt{n_{1}^{2}+n_{2}^{2}+1}}(n_{1},n_{2},1)=n\;\mathrm{on}\Gamma_{c},$$

as the normal to the crack $\Gamma_{c}$; see the description of the motivation in [42, 43].

If we substitute the relations (32) and (33) into the variational problem (1) written over the plane domain with crack $\Omega_{c}$, then the *anti-plane strain problem with limiting small strain for cracks subject to non-penetration* implies that for the given body force $f\in L^{2}(\Omega_{c};\mathbb{R})$ and boundary traction $g\in L^{2}(\Gamma_{N};\mathbb{R})$ we find the vertical displacement component $u_{3}\in H^{1}(\Omega_{c};\mathbb{R})$, its gradient $\nabla u_{3}\in L^{p\prime }(\Omega_{c};{\mathbb{R}}^{2})$, and the stresses pair $\left(\sigma_{13},\sigma_{23})\in L^{p}(\Omega_{c};{\mathbb{R}}^{2}\right)$ such that

(34a) $$〚u_{3}〛\geq 0\;\mathrm{on}\Gamma_{c},\;u_{3}=0\;\mathrm{on}\Gamma_{D},$$

(34b) $$\begin{array}{c} \frac{1}{2}\int_{\Omega_{c}}(\sigma_{13},\sigma_{23})\cdot \nabla (\bar{u}-u_{3})\mathrm{dx}\geq \int_{\Omega_{c}}f(\bar{u}-u_{3})\mathrm{dx}+\int_{\Gamma_{N}}g(\bar{u}-u_{3})dS_{x}\\ \mathrm{for all}\;\bar{u}\in H^{1}(\Omega_{c};\mathbb{R})\;\mathrm{such that}\;\bar{u}=0\;\mathrm{on}\;\Gamma_{D},〚\bar{u}〛\geq 0\;\mathrm{on}\;\Gamma_{c},\mathrm{and}\;\nabla \bar{u}\in L^{p\prime }(\Omega_{c};{\mathbb{R}}^{2}), \end{array}$$

(34c) $$\frac{1}{2}\nabla u_{3}=\Psi (\sigma ):=\frac{1}{2\mu {\left[1+\kappa {\left(2\sigma_{13}^{2}+2\sigma_{23}^{2}\right)}^{s/2}\right]}^{1/s}}(\sigma_{13},\sigma_{23}).$$

The approximation and solvability results hold for the anti-plane strain crack problem (34) as a particular case of the nonlinear crack problem (1).

In the above-formulated plane strain and anti-plane strain problems with limiting small strain for cracks subject to non-penetration, explicit singularities expressed by the stress intensity factors at the crack tip would be of primary importance for fracture applications and might be the subject of future investigation.

We note that, in the works by the authors of [9], inner regularity of the solution to limiting small strain problem under specific boundary conditions is established in convex domains, which guarantees the weak formulation in special cases. However, this result needs smooth extension outside the domain, and thus is not applicable to the case of cracks.
